# Grape peel (Syrah var.) jam as a polyphenol‐enriched functional food ingredient

**DOI:** 10.1002/fsn3.981

**Published:** 2019-04-12

**Authors:** Frederico Lopes Amorim, Mariana Barros de Cerqueira Silva, Marina Gonçalves Cirqueira, Roseane Santos Oliveira, Bruna Aparecida Souza Machado, Raquel Gutierres Gomes, Carolina Oliveira de Souza, Janice Izabel Druzian, Ederlan de Souza Ferreira, Marcelo Andrés Umsza‐Guez

**Affiliations:** ^1^ Federal University of Bahia (UFBA) Salvador Brazil; ^2^ Institute of Chemistry Sao Paulo State University (UNESP) Araraquara Brazil; ^3^ Technology College National Service of Industrial Learning (SENAI/CIMATEC) Salvador Brazil; ^4^ Department of Food Engineering State University of Maringa (UEM) Maringá Brazil

**Keywords:** antioxidant potential, bioactive compounds, grape product, physicochemical properties, shelf life

## Abstract

In this study, we evaluated the effects of the storage time on the physicochemical properties, bioactive compound content, and antioxidant capacity of jam prepared from grape peel extract to explore its potential as a supplementary food and/or functional ingredient. The ethanolic extract from Syrah var. grape peel exhibited high bioactive compound concentrations and antioxidant activity. The jam stability (prepared with 8.9% of extract) at 14°C was evaluated at 0, 15, 30, 45, and 60 days. The jam was found to contain high concentrations of polyphenolic compounds (137.0 ± 3.2 mg of gallic acid equivalent/100 g), total flavonoids (128.5 ± 23.0 mg of equivalent/100 g), and total anthocyanins (92.5 ± 4.0 mg of cyanidin equivalent/100 g). However, a large reduction in the flavonoid (70%–90%), anthocyanin (29%–35%), and phenolic (23%–30%) content was observed during storage. The free radical‐scavenging activity (DPPH^−^), ferric reducing antioxidant power (FRAP), and β‐carotene‐linoleic acid assays revealed the great antioxidant potential of the jam prepared from grape peel extract, which exhibited significant levels of radical‐neutralizing activity, especially as determined by the DPPH method with EC_50_ values ranging from 2.3 ± 0.1 to 3.9 ± 0.1 µg/ml. High *R* ^2^ values (*p *> 0.90) were obtained for the correlation between the DPPH results and the concentrations of the compounds of interest. In summary, the high bioactive compound contents and antioxidant capacity of the jam produced from grape peel suggest that it may provide health benefits as a source of natural antioxidants upon incorporation to several food industry products.

## 1 INTRODUCTION

Grape (*Vitis* spp.) is one of the most valued fruits consumed and industrialized in the world (García‐Lomillo & González‐SanJosé, 2017). According to the FAO (2014), 50 million tons are produced worldwide every year, with 75% of it being used in wine‐making. The solid waste generated by the wine industry reaches 20% of the total biomass, consisting of peels, seeds, and stalks, which may total up to 10 million tons of grape waste annually. The peel residues are considered a good source of active polyphenolic compounds, such as flavonoids, anthocyanins, phenolics, and resveratrol (Lafka, Sinanoglou, & Lazo, 2007; Martins, Roberto, Blumberg, Chen, & Macedo, 2016; Pintać et al., 2018; Vatai, Škerget, & Knez, 2009). Due to the growing interest in foods containing high levels of bioactive compounds with health benefits, along with the need for sustainable technologies, the use of by‐products has become an increasing challenge, particularly for the food industry and those working with grape pomace, due to the significant environmental impact caused by industrial activities (Caldas et al., 2018; Martins et al., 2016; Peixoto et al., 2018). In this context, the region of São Francisco Valley (the region between Petrolina and Juazeiro, Brazil) houses one of the major producers of Syrah variety grape in Brazil. This region has unique climatic characteristics, such as a semi‐arid tropical climate with average annual temperatures of ~26°C, being the only region in the world that produces grapes all year round (Aleixandre, Aleixadre‐Tudó, Bolaños‐Pizarro, & Aleixandre‐Benavent, 2016).

A variety of uses have been developed for these by‐products, either by using the whole residue (peel, seeds, and pulp), only the peels (fresh or dehydrated), or only extracts (conventional extraction with solvents or supercritical extraction; Caldas et al., 2018; García‐Lomillo & González‐SanJosé, 2017; Peiretti, Masoero, & Tassone, 2017; Peixoto et al., 2018; Pintać et al., 2018; Vatai et al., 2009; Vodnar et al., 2017). Such increasing interest is justified by the rich diversity of bioactive compounds found in these by‐products (Elkhatim, Elagib, & Hassan, 2018; Igual, García‐Martínez, Camacho, & Martínez‐Navarrete, 2013; Lafka et al., 2007). Thus, their properties and potential as food supplements have been extensively studied (Shahidi & Ambigaipalan, 2015; Xu, Burton, Kim, & Sismour, 2016). In this context, the bioactive compounds found in grape pomace/extract/products have been evaluated in terms of their nutraceuticals properties, as well as extensive searchers for scientific evidence of their performance as low‐density lipoprotein oxidation inhibitors, antimutagenics, antivirals, anti‐tumorals, and in chemoprevention by inhibiting reactions that increase the risk of coronary heart disease, reducing significant and harmful forms of several diseases, and neutralizing and/or preventing degenerative processes such as cancer, cardiovascular diseases, diabetes, oxidative stress skin damage, and others (Gül, Acun, & Sen,¸ H., Nayir, N., & Türk, S., 2013; Vodnar et al., 2017; Xu et al., 2016).

Indeed, some studies have shown the use of grape peel in products as a source of phenolic compounds, such as cereal products (cereal bars, muffins, biscuits), bread, dairy products (cheese, yogurt), as well as seafood, purees, and infusions (García‐Lomillo & González‐SanJosé, 2017), while grape peel extracts can be used in beverages and dehydrated fruit as a functional supplement to increment their phenolic content (Pedroza, Carmona, Pardo, Salinas, & Zalacain, 2012; Rózek, Achaerandio, Güell, López, & Ferrando, 2010). Some authors have demonstrated the versatility of grape pomace extracts as preservatives (antioxidant activity) in meat products (García‐Lomillo & González‐SanJosé, 2017; Shirahigue et al., 2011) and/or as antimicrobials against different foodborne pathogens (Xu et al., 2016). In many cases, the incorporation of by‐products/extracts increases the acceptability of products by the consumers due to sensory repercussion, such as color, odor, bitter and astringent taste perception, antipolyphenol oxidase activity, reduction of residual nitrites, and nitrosamines (Acun & Gül, 2014; García‐Lomillo & González‐SanJosé, 2017; Shirahigue et al., 2011).

Therefore, the aim of this study was to evaluate the effects of the storage time on the physicochemical properties, bioactive compound content, and antioxidant capacity of jam prepared from grape peel extract to explore its potential as a supplementary food and/or functional ingredient.

## 2 MATERIALS AND METHODS

### 2.1 Collection and preparation of material

Samples of grape peel of the Syrah variety were kindly provided by wineries of São Francisco Valley and transported in a plastic container at 14°C to the Food Science Laboratory of the Faculty of Technology (National Service of Industrial Training—SENAI/CIMATEC, Salvador, Brazil). The peel was manually separated from the other parts (seeds and stems), dried in an air‐circulation oven (40°C/12 hr), and immediately ground (to 125 mm sieve) to obtain a uniform particle size. The material (grape peel flour) was packed and stored under freezing conditions (−20°C) until use.

### 2.2 Grape peel extract (GPE) flour and preparation of polyphenol‐enriched jam

**Figure 1:**
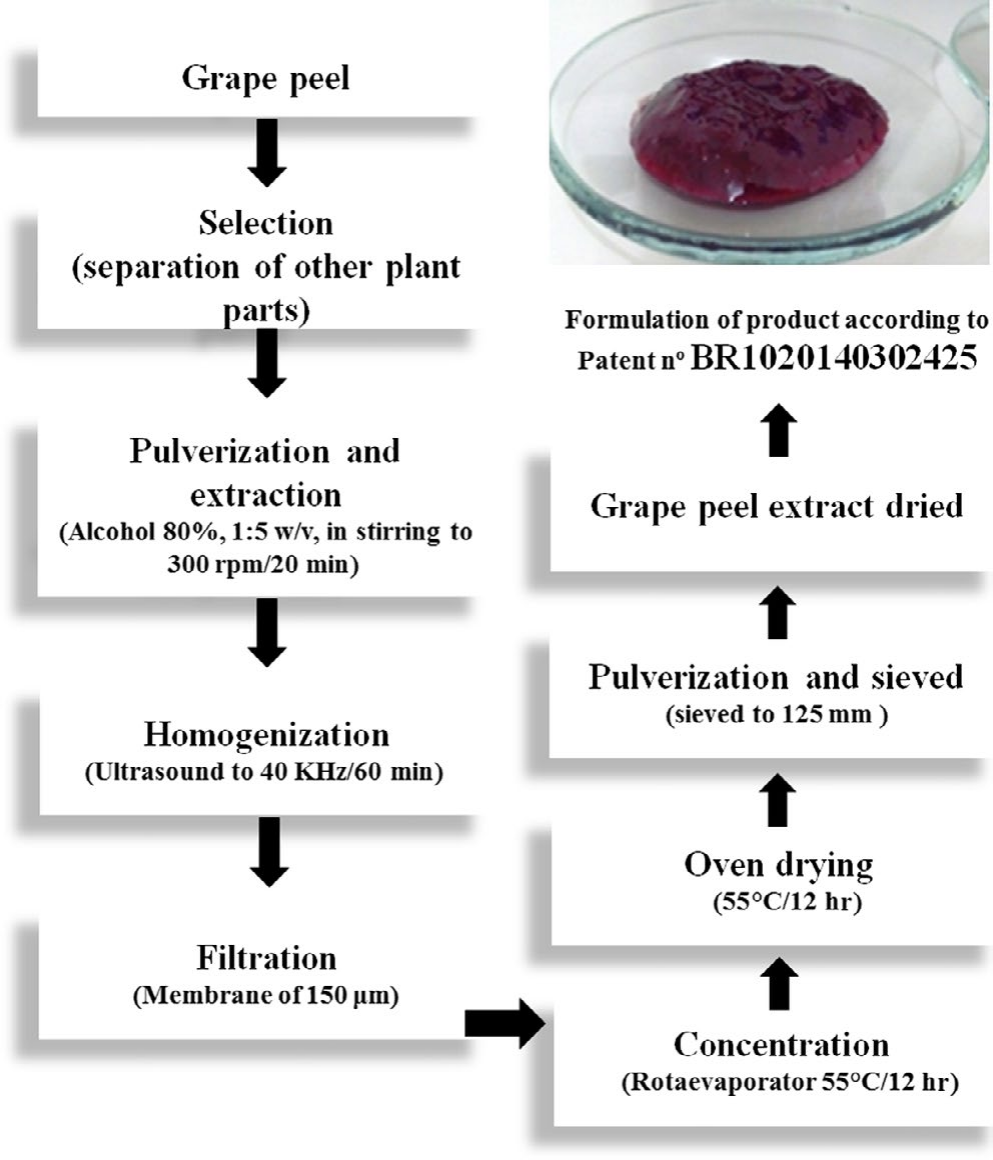
Steps for the preparation of GPE from Syrah var

The jam containing the dried grape peel extract flour was prepared according to Machado et al. (2014). The steps and conditions to obtain the GPE flour are briefly shown in Figure 1. The procedure consisted of the extraction of bioactive compounds from the grape peel flour (1:20, w:v) with alcohol solution (80%) under stirring (300 rpm) for 20 min at room temperature. The suspension (extract) was homogenized using ultrasound (40 KHz/60 min, RMS model, QUIMIS, Diadema, Brazil), filtered (0.150 µm), concentrated using the rotary evaporator (55°C, TE‐210 model, TECNAL, Piracicaba, Brazil), dried in an oven with air circulation (55°C/12 hr), and then, the material was milled and nylon sieved (125 mm). The dried grape peel extract was used in preparation of polyphenol‐enriched jam.

The jam formulation ingredients consisted of GPE 8.9 g/100 g, sucrose 36.3 g/100 g, pectin 1.15 g/100 g, citric acid 0.45 g/100 g, and water 53 ml/100 g. GPE and sugar were first mixed with water and heated to 85°C, followed by addition of the other minor ingredients. Then, this temperature was maintained for 10 min to generate the final product. The jam was stored in glass jars. Some samples were directly analyzed for the quantification of proximate composition, fatty acid profile and rheology, and the other samples were kept in an incubator (BOD MA415, Marconi, Brazil) at a controlled temperature (14°C) and were subsequently analyzed (physicochemical properties, color, bioactive compound content, and antioxidant activity) after 15, 30, 45, and 60 days. The pectin and citric acid reagents were purchased from Sigma‐Aldrich (Sigma‐Aldrich^®^ Co., St. Louis, MO, USA).

### 2.3 Analytical procedures

#### 2.3.1 Proximate composition

The proximate composition (moisture, total protein, ash, total lipid, crude fiber, and total carbohydrates) is given in g/100 g of jam in wet basis. These analyses, as well as the fatty acid profile determination, were carried out according to methods established by the AOAC (2012).

The moisture content was determined by drying at 105°C for 6 hr. The total protein content was calculated by multiplying the total nitrogen content obtained by the Kjeldahl method by the coefficient 5.30. The ash content was obtained by burning the sample in a muffle furnace at 550°C. The total lipid content was determined by gravimetric analysis after extraction in a Soxhlet device using petroleum ether for 6 hr. The crude fiber amount was determined through acid (1.25% sulfuric acid) and alkaline (1.25% sodium hydroxide) sequential treatments. The carbohydrate content was estimated by calculating the percent remaining after all the other components had been measured (% carbohydrates = 100 − [moisture + protein + lipid + ash]). The calcium, sodium, and potassium contents were quantified by atomic absorption spectroscopy with an autosampler (Model Digimed‐62, Photometer Flame^®^, São Paulo, Brazil). The samples were gravimetrically weighed and digested according to the instrument manufacturer. The samples were analyzed in triplicate and always injected in duplicate in the spectrometer; differences below 10% were considered acceptable. The results are expressed in mg/100 g of dry weight.

#### 2.3.2 Fatty acid profile

The fatty acid profile was determined by gas chromatography with a flame ionization detector (GC‐FID) as follows: the lipids were extracted using a mixture of chloroform/methanol/water (1:2:0.8 v/v/v) according to the Bligh–Dyer method (1959). The fatty acids were then transmethylated by treatment with hexane boron trifluoride and finally analyzed in a gas chromatography (Clarus 680, PerkinElmer^®^, MA, USA).

The analysis parameters included a fused silica capillary column DB‐FFAP (30 m × 0.32 mm × 0.25 mm), injector and detector temperatures of 250 and 280°C, respectively. The following thermal program was used as reported by Nascimento et al. (2013). Injections (1 μl) were performed in duplicate for each extraction. The fatty acid methyl esters were identified by comparing their retention times with those of the standards (C4‐C24, 18,919‐AMP, Sigma‐Aldrich^®^). The peak areas were determined using the Clarus Chromatography Workstation software to normalize the percentage areas of the total fatty acids. The results are given as the average of three replicate extractions.

### 2.4 Rheology study

Rheological measurements were performed in triplicate using a cone–plate rheometer (MARS III model, ThermoScientific^®^, MA, USA) at 25°C. A shear stress increase was obtained by increasing the rotation through continuous variation of the cone angular speed. Shear gradients from 0 to 700/s were applied for 30 s, with a C60 cone–plate and 0.5 ml of the sample. Thus, ascending and descending curves were obtained. The strain rate was determined with a computer program (RheoWin Data Manager, ThermoScientific^®^) using equations A1 and A2:

A1γ=ω/sinθ

$$A2\;\tau =\frac{T}{\frac{2}{3}\pi r^{3}}$$

where *γ* is the shear rate (1/s), *T* denotes the shear stress (Pa), *w* refers to the angular speed of the cone (rpm), and *π* is the cone angle. The rheological behavior description was performed using the Power Law rheological model (*K* = *tγ^n^*) with the aid of the Origin software, version 6.0.

### 2.5 Jam stability and shelf life

The jam was kept in an incubator (BOD MA415, Marconi, SP, Brazil) at a controlled temperature (14°C) and was subsequently analyzed after 0, 15, 30, 45, and 60 days. The stability of the product was evaluated in terms of the physicochemical properties, color, bioactive compound content (total phenolics, total flavonoids, and total anthocyanins), and antioxidant activity (as determined by DPPH, FRAP and β‐carotene/linoleic acid assays).

#### 2.5.1 Physicochemical properties

The water activity (*A*_w_) was determined using a Labmaster AW NEO (Novasina^®^, Lachen, Germany) instrument. The potential hydrogen (pH) was determined by potentiometry using a pHmeter DM‐22 (Digemed^®^, São Paulo, Brazil) at 20°C, and the titratable acidity (TA, g 100/g citric acid) by titration analysis. Total soluble solid (TSS) content (Brix) was measured using a Brix temperature‐compensating refractometer (HI 96801, Hanna Instruments^®^, RI, USA).

#### 2.5.2 Color parameters

The jam color was evaluated using the CIE Lab parameters of lightness (L*), redness/greenness color coordinate (a*), and yellowness/blueness color coordinate (b*), using a 2‐mm path length cuvette in a colorimeter CR‐400 (Konica Minolta^®^, Tokyo, Japan) following the manufacturer's recommendations.

#### 2.5.3 Total phenolic content

The total phenolic content was determined spectrophotometrically (Beckman^®^ Coulter DU‐70 UV/VIS Spectrophotometer, CA, USA) according to the Folin–Ciocalteu method (Slinkard & Singleton, 1977) through the absorbance measured at 765 nm. A calibration curve was built using standard gallic acid (25–200 µg/ml) affording the equation *y* = 0.0073*x* – 0.0591 (*R* ^2^ = 0.9994). The results are expressed as mg of gallic acid equivalent/100 g of jam fresh weight (mg GAE/100 g).

#### 2.5.4 Total flavonoid content

The total flavonoid content was determined by the aluminum chloride colorimetric method as described by Chang, Yang, Wen, and Chern (2002), using the absorbance measured at 415 nm (Beckman^®^ Coulter DU‐70 UV/VIS Spectrophotometer). A calibration curve was constructed from the standard quercetin (5–35 µg/ml) affording the equation *y* = 0.0287*x* − 0.0076 (*R* ^2^ = 0.9996). The results are expressed as mg of quercetin equivalent/100 g of jam fresh weight (mg QE/100 g).

#### 2.5.5 Total anthocyanin content

The anthocyanin content was determined according to the method proposed by Lees and Francis (1972), using the absorbance measured at 535 nm (Beckman^®^ Coulter DU‐70 UV/VIS Spectrophotometer). The total anthocyanin content was obtained using the equation: Total anthocyanin content mg/L = (*A* × *FD*)/(*ε* × *b*), where *A* is the absorbance (535 nm), *ε* denotes the absorbance coefficient of cyaniding‐3‐glucoside (26,900, MW 449.2), *b* corresponds to the thickness of the cuvette (1 cm), and *FD* refers to the dilution factor of the extract. The results are expressed as mg/100 g of jam fresh weight.

#### 2.5.6 Free radical‐scavenging activity – DPPH method

The free radical‐scavenging activity was evaluated following the method proposed by Brand‐Williams, Cuvelier, and Berset (1995), where the decrease in the absorbance at 515 nm of 100 mmol/L DPPH dissolved in 80% methanol is measured 30 min after the sample addition. The antioxidant activity of the jam (fresh weight) is expressed in EC_50_ µg/g.

#### 2.5.7 **Ferric reducing antioxidant power**—**FRAP method**

The ferric reducing antioxidant power was evaluated according to the method proposed by Arnous, Makris, and Kefalas (2002), which is based on the direct measurement of the antioxidant (reducing) ability through the reduction of the complex Fe^3+^/tripyridyltriazine (TPTZ) to Fe^2+^ under acidic pH (3.6). The reducing capacity of the jam was determined using the absorbance at 620 nm (Beckman^®^ Coulter DU‐70 UV/VIS Spectrophotometer). The results are expressed as µmol/g of trolox equivalent of the jam fresh weight.

#### 2.5.8 Co‐oxidation of the β‐carotene/linoleic acid system

The oxidation inhibition power was determined by the method described by Miller (1971), based in the evaluation of the decoloration of the β‐carotene/linoleic acid system. The mechanism of β‐carotene bleaching is a free radical‐mediated process resulting from the presence of hydroperoxides formed from linoleic acid, which can be monitored spectrophotometrically at 470 nm (Beckman^®^ Coulter DU‐70 UV/VIS Spectrophotometer). The assay was performed using a standard concentration of the β‐carotene/linoleic acid system and extract/trolox at different concentrations. The results are expressed as the percentage (%) of oxidation inhibition relative to that of the antioxidant standard.

### 2.6 Statistical analyses

All analyses were performed in triplicate, and the results are presented as mean ± standard deviation (*SD*). The data were subjected to analysis of variance (ANOVA), and the results with significant differences were used in a Tukey test for multiple comparison of the means at a significance level of 5% using the SigmaStat^®^ software.

## 3 RESULTS AND DISCUSSION

### 3.1 Chemical and nutritional composition of the jam

The aim of this study was to create value‐added product—jam polyphenol‐enriched of low calorie produced from grape peel dried extract. Thus, the proximate composition (moisture, ash, total protein, total lipids, fatty acid composition, fiber, and carbohydrates), energy value and content of some individual mineral elements of the jam are presented in Table 1.

**Table 1:** Proximate composition of the jam prepared from grape peel dried extract

Parameters^a^	Jam (g/100 g)
Moisture	53.28 ± 0.50
Total ash	0.29 ± 0.27
Sodium (mg/100 g)	3.60 ± 0.15
Potassium (mg/100 g)	4.50 ± 0.28
Calcium (mg/100 g)	6.60 ± 0.27
Protein	1.21 ± 0.39
Total lipids	0.03 ± 0.00
Total carbohydrates^b^	45.19 ± 0.22
Dietary fiber	2.02 ± 0.17
Energy (Kcal)	177.8 ± 8.23

a The values (means ± *SE*) correspond to averages from three replicates.

b Defined by the difference between 100 and the sum of the percentages of others components, as shown in the material and methods.

The jam prepared had the highest moisture content (53%) when compared to other formulations (31%–34%), which on foods, generally a high value of moisture content indicates a smaller shelf life. On the other hand, the jam had a lower total carbohydrate content (45.19 ± 0.22%) and lower energy value (177.8 kcal/100 g) when compared to commercial jams (65–67% and 266–273 kcal/100 g, respectively) (Naeem et al., 2017). These findings were possible because of the minor amount of the dried extract used in the formulation (8.9%). Although the protein (1.21%) and ash (0.29%) contents of the jam were higher compared to the content of whole fruit jams, such as apricot, blueberry, grape, and strawberry (protein of 0.27%–0.43% and ash of 0.12%–0.25%), respectively (Naeem et al., 2017). All of the common ingredients used in the formulation of jams are not an abundant source of protein and ash; however, the differences in these parameters are related to the specific characteristics of the species (Touati, Tarazona‐Díaz, Aguayo, & Louaileche, 2014).

Other studies of grapevine by‐products in the literature have provided very little information on the fatty acid content. However, these viticulture residues may have potential for several alternatives uses (Hussein & Abdrabba, 2015). For example, the waste grape is widely used in animal feeds as organic fertilizer (Caldas et al., 2018) and to release of color, phenolic, and aroma compounds into juice and wine (Pedroza et al., 2012). Other alternative uses of the viticulture waste are the extractives represented by classes of organic compounds such as polyphenols and triterpenic acids which have pharmaceutical, cosmetic, and nutritional applications. In addition, it has been used in production of bacterial cellulose, biofuel (bioethanol) alternative to petrofuels, and new foods products with functional appeal (Peixoto et al., 2018).

Table 2 shows the fatty acid profile of the jam. The fatty acid with the highest concentration is palmitic acid (26.4%), followed by linoleic acid (21%), and oleic acid (20.9%), amounting to 77% of the total fatty acids. Previous research has shown that, in the peel of Nebbiolo, Barbera, Syrah, Grenache, Pinot Noir, and Cabernet Sauvignon grapes, the predominant fatty acids are palmitic, oleic, and linoleic, representing approximately 80% of the total composition (Peiretti et al., 2017; Vodnar et al., 2017). However, some varieties show higher contents of unsaturated fatty acids, especially linoleic acid, α‐linolenic acid, and oleic + cisvaccenic acids. Recent studies have shown that such enhanced synthesis of unsaturated fatty acids occurs due to an increase in the abiotic stress experienced by the grapevines, usually caused by mechanical damage (Chitarrini et al., 2017). The importance of polyunsaturated fatty acids (oleic and linoleic acid) to the human body has already been established; they are essential in small quantities in the human body as they cannot be synthesized (Hussein & Abdrabba, 2015; Rózek et al., 2010; Shirahigue et al., 2011). Therefore, the prepared product is a valuable source of PUFAs, which are associated with health benefits for the consumers.

**Table 2:** Fatty acid composition (%) of the jam prepared from grape peel dried extract

Fatty acids	% of total fatty acid^a^
Butyric acid (C4:0)	3.28 ± 0.31
Capric acid (C10:0)	0.45 ± 0.02
Lauric acid (C12:0)	0.87 ± 0.04
Myristic acid (C14:0)	0.66 ± 0.03
Palmitic acid (C16:0)	26.37 ± 1.32
Palmitoleic acid (C16:1 ∆^7^ cis) ω−9	6.19 ± 0.31
Oleic acid (C18:1 ∆^9^ cis) ω−9	20.88 ± 1.04
Linoleic acid (C18:2 ∆^9,12^ cis) ω−6	20.96 ± 1.05
α‐Linolenic acid (C18:3 ∆^9,12,15^ cis) ω−3	6.63 ± 0.33
Erucic acid (C22:1 ∆^13^ cis) ω−9	1.69 ± 0.08
Docosadienoic acid (C22:2 ∆^13,16^ cis) ω−6	0.59 ± 0.03
Unidentified fatty acid	11.43 ± 0.16
Total SFA	31.63
Total MUFA	28.76
Total PUFA	28.18

MUFA, monounsaturated fatty acids; PUFA, polyunsaturated fatty acids; SFA, saturated fatty acids.

a The values (means ± *SE*) correspond to averages from three replicates.

From the results of the rheological characterization, that is, the flow behavior index, the jam is classified as a non‐Newtonian fluid because the value for the flow index (*n*) is smaller than 1.0 (data not shown). It was observed that the *K* index increased during the experiment, which may be explained by migration of water to the surface, resulting in a more firm product. Also, the increased *K* index may be linked to the presence of phenolic compounds, anthocyanins, and other gelling compounds present in the grape peel (Fester, Slatter, & Alderman, 2012).

### 3.2 Physicochemical stability during storage

In the present study, the effect of storage time (15, 30, 45, and 60 days) at 14°C on the physicochemical properties, bioactive compound content, and antioxidant capacity of jam prepared from grape peel dried extract were investigated.

#### 3.2.1 Effect of storage time on Aw, pH, TA and TSS

The storage time from food formulations can affect their sensory, structural, and nutritional properties. It is well known that the water activity and pH are essential parameters for the maintenance of these properties (Mazur et al., 2014; Touati et al., 2014). Therefore, the water activity (Aw), potential hydrogen (pH), titratable acidity (TA), and total soluble solid (TSS) of jam were determined (Table 3).

**Table 3:** Aw, pH, titratable acidity, and total soluble solids during storage of the jam prepared from grape peel dried extract

Parameters^b^	Period (time in days)				
	0	15	30	45	60
Activity water (Aw)	0.93 ± 0.40	0.96 ± 0.30	0.93 ± 0.36	0.95 ± 0.50	0.92 ± 0.32
pH	3.40 ± 0.16	3.51 ± 0.23	3.25 ± 0.34	3.86 ± 0.28	3.91 ± 0.17
Titratable acidity (g 100/g of citric acid)	1.20 ± 0.24	0.75 ± 0.10	1.43 ± 0.09	1.33 ± 0.16	1.33 ± 0.03
Total soluble solids (ºBrix)	43.02 ± 0.12^a^	40.05 ± 0.20^b^	39.50 ± 0.11^b^	39.00 ± 0.09^b^	40.60 ± 0.33^b^

a Different letters in the same line indicate significant differences between the values (*p* < 0.05).

b The values (means ± *SE*) correspond to averages from three replicates.

Despite the Aw value of the product could be considered to be a high one (up of 0.9), conducing to the growth of microorganisms and chemical and biological reactions, the TA (0.75–1.43 g/100 g of citric acid) values are considered safe, since the acidity level protects against the development of microorganisms (Touati et al., 2014). There were no statistically significant differences among the storage time on the Aw, pH, and TA values. Moreover, those parameters were similar to those found for low‐calorie jams (0.90–0.96, 3.50–3.57, and 0.30–0.41; Belović, Torbica, Pajić‐Lijaković, & Mastilović, 2017) and grapefruit jam (0.92–0.95, and 3.39–3.40) (Igual et al., 2013), respectively.

The TSS changes were significant (*p* < 0.05) under storage time, where it has decreased 5.62% (60 days), compared to the initial value. It is suggested that this difference may be related to an increase in the acidity, resulting in the conversion of sugars present in organic acids (Silva, Viana, Silva, Oliveira, & Gomes Filho, 2008), but the results found in this work, ranging between 39.5 and 43 ºBrix, were within values reported by Belović et al. (2017) for jams prepared from tomato pomace of low calorie which ranked between 24 and 48 ºBrix, but it was smaller than those found by Touati et al. (2014) for commercial apricot jam (64%).

#### 3.2.2 Effect of storage time on color

**Figure 2:**
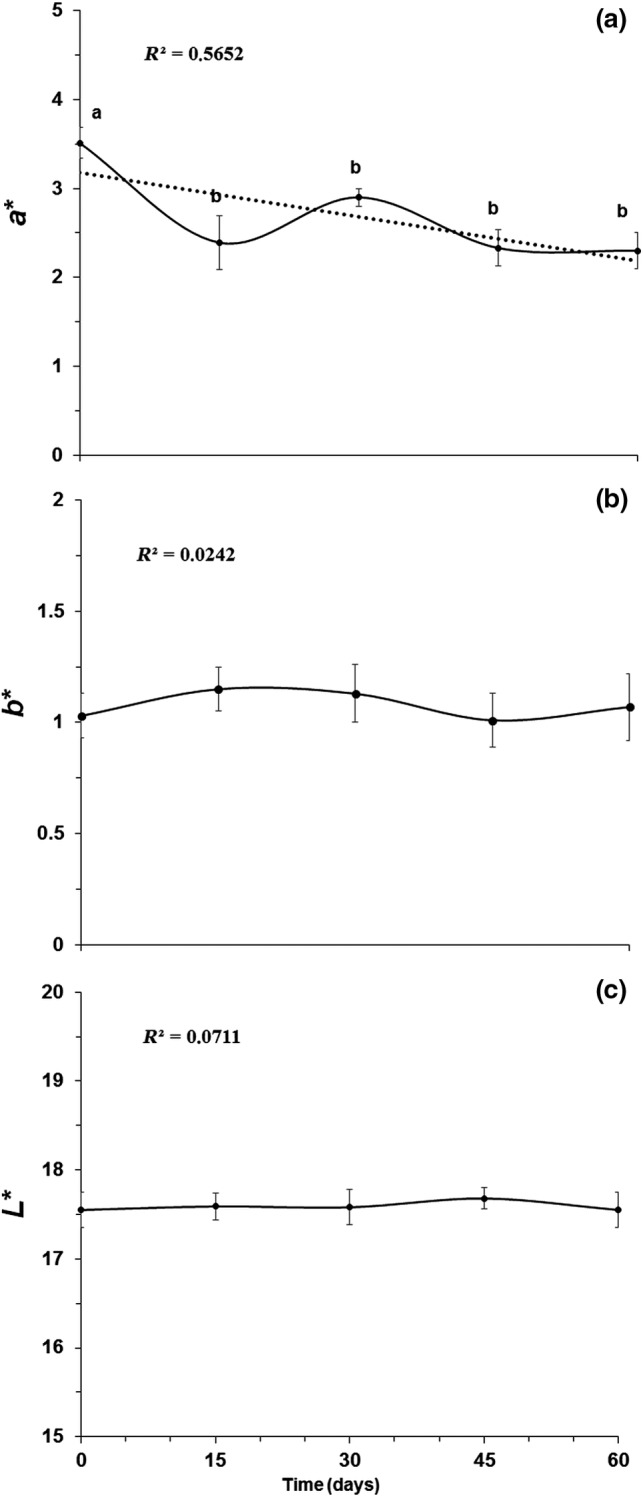
Evaluation of the three‐dimensional color coordinates a* (A), b* (B), and L* (C) during storage of the jam prepared from grape peel dried extract. ^‡^The values (means ± *SE*) correspond to averages from three replicates. Different letters in the same line indicate significant differences between the values (*P* < 0.05)

Figure 2 shows the changes in color parameters of the low‐calorie polyphenol‐enriched jam produced from grape peel dried extract, where it is observed that the b* (Figure 2b) and L* (Figure 2c) indexes do not show significant differences (*p *> 0.05) during storage by 60 days at 14°C, as confirmed by the low correlation values (*R* ^2^ = 0.0242–0.0711) between the storage time and these parameters. This is because the characteristic pigmentation (yellow/blue) related to these parameters is not a major constituent in this grape variety, especially for products elaborated from the peel. The results observed were in accordance with the ones reported by Mazur et al. (2014) that did not find any significant influence on L*, chroma and hue values of the jam stored during 6 months at 4 and 20°C. However, Touati et al. (2014) showed that the color attributes of apricot jam were influenced by time (20, 40 and 60 days) and temperature (5, 25 and 37°C) factors. The authors found a decreased of L* value 14.5%, 16.8%, and 20.1% of apricot jam stored at 5, 25, and 37°C by 60 days.

**Figure 3:**
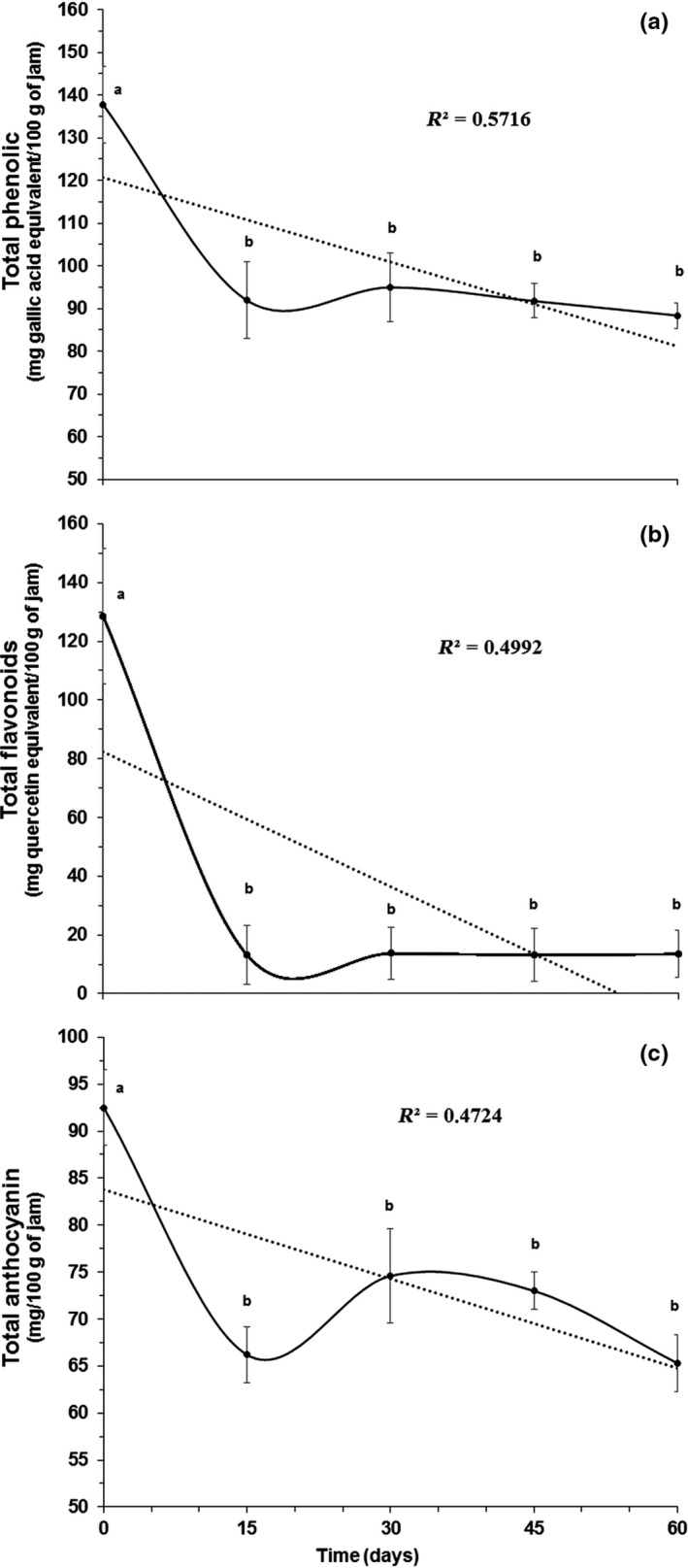
Total phenolic (a), flavonoid (b), and anthocyanin (c) contents during storage of the jam prepared from grape peel dried extract. ^‡^The values (means ± *SE*) correspond to averages from three replicates. Different letters in the same line indicate significant differences between the values (*P* < 0.05)

On the other hand, the product displays high values for the coordinate a* (3.52 ± 0.17) at short storage times, with intense purple color (Figure 2a). A decrease (−37%, *p *< 0.001) of the a* value can be observed after 60 days (*R* ^2^ = 0.5652), and this variation may be the result of pH changes and possible degradation/oxidation of some red/violet pigments, which are responsible for the color of the product (Lee, Lee, Lee, Lee, & Kim, 2013; Pedroza et al., 2012). In part, this result is directly related to the decline in the total anthocyanin (−30%, *p < *0.001) and total flavonoid (−90%, *p < *0.001) concentrations, as shown in Figure 3.

Color changes were also observed in a study with wine must produced at different times of the year and in packages containing extracts of anthocyanins obtained from grape by‐products and blueberry jam (Idham, Muhamad, & Sarmidi, 2012; Lafarga, Aguiló‐Aguayo, Bobo, Chung, & Tiwari, 2018). In addition, previous studies have already demonstrated an intense reduction of the color parameters in extracts obtained from peel of three grape varieties as a function of the time from packing, especially for the coordinate a* (Vatai et al., 2009). Anthocyanins exhibit different color shades depending on the pH and may have intense red color at pH values below 3.5, losing color intensity until they become colorless with the increasing pH, especially at values >5.0. The chemical characteristics of anthocyanins (polymerization, copigmentation, and acetylation) affect the color properties: intensity, hue, and stability. The stability can also be influenced by the presence of light and/or O_2_ during processing, heat treatment, and storage of the product (Hussein & Abdrabba, 2015; Idham et al., 2012; Kim & Padilla‐Zakour, 2004). Nevertheless, the stability of this parameter is critical for a market product, since the food color is considered one of the most important aspect to which consumers are sensitive at the time of purchase and consumption choice (Touati et al., 2014).

### 3.3 Total phenolic, flavonoid, and anthocyanin contents during storage

Figure 3 shows the total phenolic, total flavonoid, and total anthocyanin contents of the jam polyphenol‐enriched of low calorie produced from grape peel dried extract during storage for 60 days at a controlled temperature of 14°C. In this study, the contents found in the dried extract were 3,346.0 mg GAE/100 g for total phenolics, 3,107.7 mg QE/100 g for total flavonoids, and 2,223.2 mg/100 g for total anthocyanins (dry weight). In other studies that evaluated the total phenolic concentration in several varieties of grape peels, similar values were observed to those obtained in this study (Makris, Boskou, & Andrikopoulos, 2007; Martins et al., 2016; Pedroza et al., 2012; Rockenbach, Silva, Rodrigues, Kuskoski, & Fett, 2011; Vodnar et al., 2017)

The concentrations of total phenolic compounds (90 mg GAE/100 g), total flavonoids (20 mg QE/100 g), and total anthocyanins (65 mg/100 g) in the jam (final product) were highest compared to those of other by‐products, despite the peel dried extract constituting only 8.9% of the final product formulation. However, a reduction of 35.7% (*p < *0.001) in the concentration of phenolic compounds was observed in the first 15 days, remaining constant afterward up to 60 days, as also observed in blueberry jellies (Lafarga et al., 2018). Mazur et al. (2014) showed that the total phenolic and total anthocyanins contents found in fresh strawberry jam of nearly ripe, ripe and fully ripe fruits of the cultivars “Blink,” “Polka,” and “Senga Sengana” were between 71 to 134 mg GAE/100 g and 7.4 to 20.5 mg/100 g, respectively. It is important to highlight that these values represent a product with 60% fruit. The authors also observed a reduction of 10.9% and 77.6% in the total phenolic and total anthocyanins concentrations in the jam stored for 3 months at 20°C. Notably, storage at higher temperatures for a longer period of time had negative influence on most of the quality parameters measured.

The quantified values of flavonoids and anthocyanins (Figure 3b,c) show a significant reduction (88% and 22%) between *T* = 0 and *T* = 1 (15 days), respectively. Previous studies have shown a rapid bioactive compounds and pigment degradation during storage of jams. Mazur et al. (2014) observed that after 3 months of storage, anthocyanins degraded 60% in jams prepared from nearly ripe fruits, 52% in jams made from ripe fruits, and 50% in jams from fully ripe fruits. After 6 months, it was observed a reduction of 85%, 75%, and 70%, respectively. In addition, the negative influence of the temperature for these compounds was shown by Pedroza et al. (2012) for four varieties of waste grape skins, where it was observed a decrease of total anthocyanins concentration between 27.9% to 30%, 79.7 to 87.7, and 92.5 to 100%, in dehydrated waste grape skins at 60, 90, and 100°C, respectively.

The reduction of the concentration of these compounds occurs because other anthocyanins compounds are less resistant to light, pH, and long storage periods (Lafarga et al., 2018; Lee et al., 2013; Martins et al., 2016). Some studies investigating natural sources to obtain anthocyanins in by‐products showed a direct relationship between the increasing Aw and the anthocyanin degradation, as observed in elaborated products from grape peel extract (Anastasiadi, Pratsinis, Kletsas, Skaltsounis, & Haroutounian, 2012; Rockenbach, Gonzaga et al., 2011). The water activity presented an average value of 0.923, which can be considered high, with a concomitant decrease in the anthocyanin concentration of 29% by the end of the study (60 days).

Several studies have shown variations in the concentration of phenolic compounds present in fresh peel, extracts, peel flour, and other forms of processed residues from different grape varieties. Notably, the soil, water, climatic conditions, and region induce significant variations in these compounds (Gül et al., 2013; Makris et al., 2007; Martins et al., 2016; Rockenbach, Gonzaga et al., 2011; Rockenbach, Silva et al., 2011). These variations occur particularly in traditional jam manufacture, where all the ingredients are mixed and its concentrated by applying an intense thermal treatment (cooking process) to reach the required final soluble solid content, implying an undesirable impact in color, flavor, and nutritional and functional value of the product (Belović et al., 2017; Kim & Padilla‐Zakour, 2004; Vodnar et al., 2017). The influence of different processing methods on preservation of phenolic compounds in different products have been studied (Caldas et al., 2018; Gül et al., 2013; Lafka et al., 2007; Pedroza et al., 2012), and differences between species after processing fruits into jams have been also reported (Acun & Gül, 2014; Elkhatim et al., 2018; Howard, Castrodale, Brownmiller, & Mauromoustakos, 2010; Lafarga et al., 2018; Lee et al., 2013).

### 3.4 Antioxidant activity during storage

The antioxidant activity of the low‐calorie polyphenol‐enriched jam produced from grape peel dried extract during storage for 60 days at a controlled temperature of 14°C was evaluated by different methods is shown in Table 4. The antioxidant activity by the DPPH method presented significant differences in the first 15 days, but not in the following days. Thus, lower antioxidant activity (EC_50_ = 3.94 µg/ml) was observed at the beginning compared to that after 60 days of storage (EC_50_ = 2.29 µg/ml); this result corresponds to an increase in 42% of the antioxidant activity in the product, showing high potential for the reduction of DPPH (ranging from 20% to 40%). EC_50_ values above 250 mg/ml indicate low potential antioxidants (Xu et al., 2016). Thus, the jam itself presents as an excellent food source of bioactive compounds with antioxidant activity. Similar values of antioxidant activity have been reported by previous studies that incorporated the crude extracts from waste sources as antioxidant ingredients (Pedroza et al., 2012; Rózek et al., 2010; Shahidi & Ambigaipalan, 2015).

**Table 4:** Evaluation of the antioxidant activity during storage of the jam prepared from grape peel dried extract

Antioxidant activity^a^	Period (time in days)				
	0	15	30	45	60
DPPH (EC_50_ µg/ml)	3.94 ± 0.02^a^	2.76 ± 0.23^b^	2.62 ± 0.34^b^	2.47 ± 0.3^b^	2.29 ± 0.03^b^
FRAP (µmol/g)	396.50 ± 4.40^b^	406.00 ± 2.60^b^	427.40 ± 3.60^a^	395.00 ± 6.13^b^	390.20 ± 3.67^b^
β‐carotene/linoleic acid (%)	24.00 ± 0.33^c^	28.07 ± 0.42^b^	36.07 ± 1.42^a^	26.13 ± 0.56^bc^	25.10 ± 0.47^c^

Different letters in the same line indicate significant differences between the values (*p* < 0.05).

a The values (means ± *SE*) correspond to averages from three replicates.

Using the β‐carotene autoxidation method, the antioxidant activity at the beginning of the experiment was found to change from day 30 onward, where the product obtained from grape peel showed 24% antioxidant activity at day 0 and 34% after 30 days. However, at days 45 and 60, the antioxidant activity declined, reaching values close to the initial one. The antioxidant activity measured by the FRAP method was 396.5 μmol/g of trolox equivalent at the initial time and 427.4 μmol/g of trolox equivalent after 30 days of storage. On the following days, the final value of antioxidant activity was similar to that at the beginning of the experiment, in agreement with the results by other authors (Kim & Padilla‐Zakour, 2004; Rockenbach, Silva et al., 2011).

Due to the growing health concerns and higher incidence of degenerative diseases during the recent decades, there has been an increase in interest for low‐calorie food consumption and in diets rich in phytochemicals and antioxidants in order to perform a protective role on health and prevent diseases (Belović et al., 2017). In fact, the phytochemicals are compounds present in fruits, vegetables, grains, and other plant foods, but they are found in higher concentrations in waste food, such as peels, seeds, stems, and others by‐products, which have significant economic potential. Therefore, in the present study we show an alternative use for by‐products (peel) from the viticulture industry with a great antioxidant potential.

**Figure 4:**
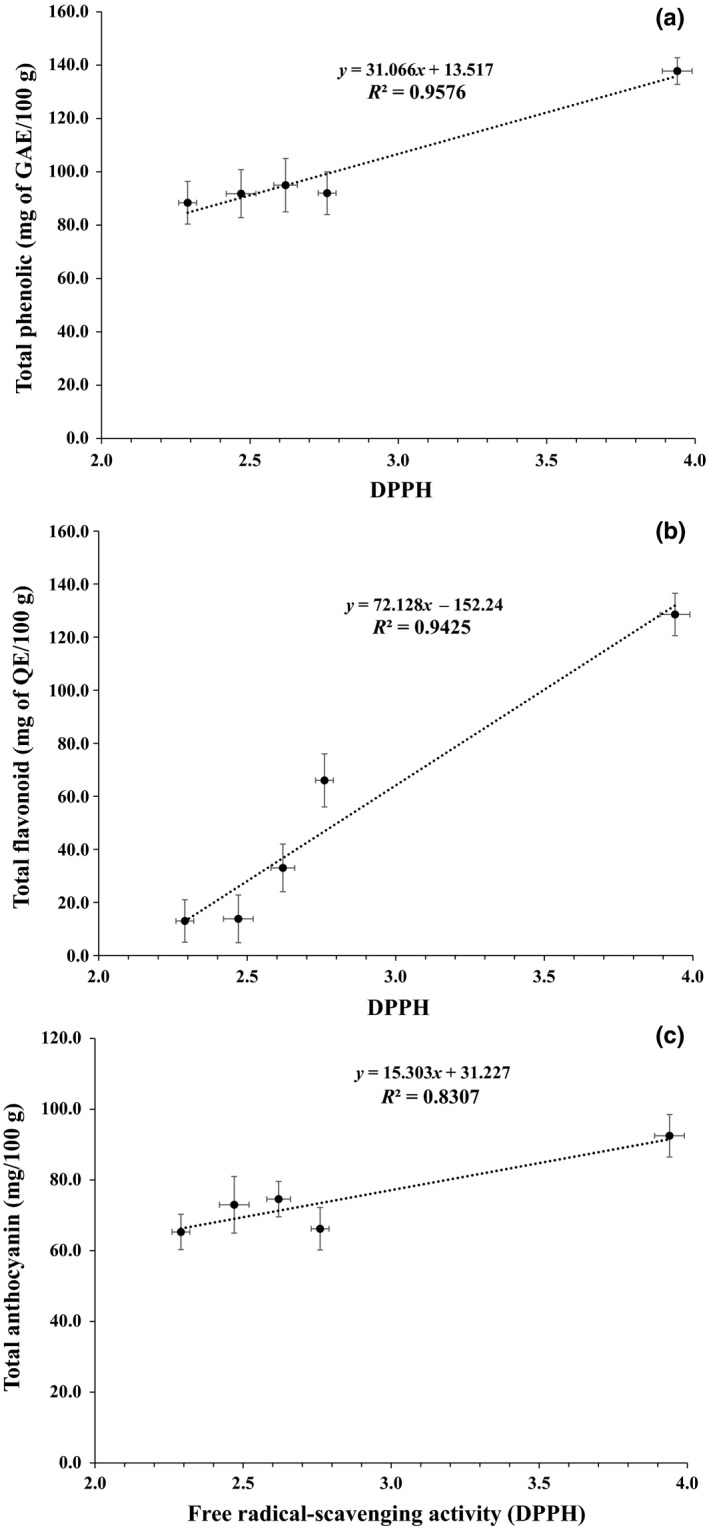
Correlation between the bioactive compound contents (total phenolic (a), flavonoid (b), and anthocyanin (c) concentrations) and the DPPH results. ^‡^The values (means ± *SE*) correspond to averages from three replicates. Different letters in the same line indicate significant differences between the values (*p* < 0.05)

Figure 4 shows the *R* ^2^ values for the correlation between the DPPH results and the total phenolic, flavonoid, and anthocyanin contents. A high correlation (*p *> 0.800) was observed between these parameters, especially with the total phenolic content (*p *> 0.950, Pearson correlation). Important correlations between phenolic compounds and the antioxidant capacity of grape residue extracts have been observed in other studies (Anastasiadi et al., 2012; Elkhatim et al., 2018; Martins et al., 2016; Xu et al., 2016). Regarding products obtained with jams produced from fruits with high concentrations of these compounds, a reduction of the phenolic concentration and antioxidant capacity of the product was observed during manufacturing (from fruit to jam), and in terms of the time and/or temperature of storage (Howard et al., 2010; Kim & Padilla‐Zakour, 2004; Lafarga et al., 2018), consistent with the results observed in this study.

## 4 CONCLUSION

In this study, the effects on the physicochemical properties, bioactive compound content, and antioxidant capacity of low‐calorie polyphenol‐enriched jam produced from grape peel dried extract stored for 0, 15, 30, 45, and 60 days at 14°C were evaluated. The jam has lower total carbohydrate content (45%) and lower energy value (177.8 kcal/100 g) when compared to commercial products. The physicochemical results indicated that the jam has good stability, but high water activity value indicating that this product might have shorter shelf life. The degradation of the pigments, especially of flavonoid and anthocyanin compounds was observed in stored jam until the 15° day, showing that had a negative influence on color, but do not decreased antioxidant activity. On the contrary, the jam presents significant amount of bioactive compounds and antioxidant activity, although only 8.9% (w/w) of the dried extract was used in the formulation. Thus, this study shows an alternative use for by‐products (peel) from the viticulture industry. The results indicate that these may provide health benefits as a source of natural antioxidants when incorporated into different food industry products.

## CONFLICT OF INTEREST

All authors declare no conflict of interest with regard to the described research, the publication of the result, and financial issues.

## ETHICAL STATEMENT

This study does not involve any humananimal testing.
